# Neuroinflammation in Alzheimer's disease wanes with age

**DOI:** 10.1186/1742-2094-8-171

**Published:** 2011-12-07

**Authors:** Jeroen JM Hoozemans, Annemieke JM Rozemuller, Elise S van Haastert, Piet Eikelenboom, Willem A van Gool

**Affiliations:** 1 Department of Pathology, VU University Medical Center, P.O. Box 7057, 1007 MB Amsterdam, The Netherlands; 2 Department of Psychiatry, VU University Medical Center, Valeriusplein 9, 1075 BG Amsterdam, The Netherlands; 3 Department of Neurology, Academic Medical Center, University of Amsterdam, P.O. Box 22660, 1100 DD Amsterdam, The Netherlands

**Keywords:** Alzheimer's disease, microglia, astrocyte, aging

## Abstract

**Background:**

Inflammation is a prominent feature in Alzheimer's disease (AD). It has been proposed that aging has an effect on the function of inflammation in the brain, thereby contributing to the development of age-related diseases like AD. However, the age-dependent relationship between inflammation and clinical phenotype of AD has never been investigated.

**Methods:**

In this study we have analysed features of the neuroinflammatory response in clinically and pathologically confirmed AD and control cases in relation to age (range 52-97 years). The mid-temporal cortex of 19 controls and 19 AD cases was assessed for the occurrence of microglia and astrocytes by immunohistochemistry using antibodies directed against CD68 (KP1), HLA class II (CR3/43) and glial fibrillary acidic protein (GFAP).

**Results:**

By measuring the area density of immunoreactivity we found significantly more microglia and astrocytes in AD cases younger than 80 years compared to older AD patients. In addition, the presence of KP1, CR3/43 and GFAP decreases significantly with increasing age in AD.

**Conclusion:**

Our data suggest that the association between neuroinflammation and AD is stronger in relatively young patients than in the oldest patients. This age-dependent relationship between inflammation and clinical phenotype of AD has implications for the interpretation of biomarkers and treatment of the disease.

## Background

Alzheimer's disease (AD) is a chronic neurodegenerative disease and is the most common cause of dementia. Two hallmarks of the disease are senile plaques, which are mainly composed of extracellular deposits of amyloid β (Aβ), and neurofibrillary tangles, which consist of intracellular aggregates of aberrantly phosphorylated tau protein. Senile plaques are associated with an inflammatory response as shown by an increased presence of activated complement proteins, cytokines, and activated microglia and astrocytes [1]. It is suggested that this inflammatory response, also referred to as neuroinflammation, plays a prominent and early role in AD [2-4]. A role for inflammation in AD has recently gained strong support from genome-wide association studies that have identified genes involved in inflammation that are associated with increased risk of developing AD [5,6].

The inflammatory response in AD is a double-edged sword. It is a self-defence reaction aimed at eliminating injurious stimuli and restoring tissue integrity. However, inflammation may become a harmful process when it becomes chronic. Chronic activation of the inflammatory response in AD produces pro-inflammatory cytokines, prostaglandins and reactive oxygen species that exacerbate Aβ deposition and induce neuronal dysfunction [7]. Despite wide acceptance of the idea that inflammation contributes to AD, it remains unclear at what stages of AD inflammation is beneficial or detrimental [8].

Clinicopathological studies suggest that neuroinflammation, and in particular microglial activation, is an early event in AD pathology. The volume of tissue occupied by microglia, the brain resident macrophages, increases with severity of dementia, but peaks in moderately affected cases [2]. The volume density of microglia is already increased in early pathological stages of AD and in cognitively normal subjects with frequent presence of plaques and tangles [3,4]. Clinical studies using positron emission tomography and the peripheral benzodiazepine ligand PK11195 as a marker for activated microglia indicate that activation of microglia occurs already in mild and early forms of AD-type dementia and precedes cerebral atrophy in AD [9,10]. These data indicate that neuroinflammation is involved at an early stage of AD pathogenesis.

It has recently been proposed that aging has an effect on the function of inflammation in the brain. Some studies have suggested that aging facilitates an imbalance between pro- and anti-inflammatory mechanisms resulting in a low grade, chronic pro-inflammatory status, referred to as inflammaging, while other studies have proposed that microglia deteriorate during aging [11,12]. These studies have suggested that the effect of aging on inflammation in the brain contributes to the development of age-related diseases like AD. However, the age-dependent relationship between inflammation and clinical phenotype of AD has never been investigated. In this study, we examined the presence of microglia and astrocytes, as markers of neuroinflammation, in clinically and pathologically confirmed AD and non-demented control cases in relation to age. Our data suggest that the association between neuroinflammation and AD is much stronger in relatively young patients as compared to the oldest patients.

## Methods

### Case selection

Post-mortem brain material was obtained from the Netherlands Brain Bank (Amsterdam, The Netherlands). All donors or their next of kin provided written informed consent for brain autopsy and use of tissue and medical records for research purposes. Between 1999 and 2001 non-demented control and AD cases were sequentially selected. Dementia status at death was determined on the basis of all information available for each case during the last year of life. Severity of dementia was measured using the Global Deterioration Scale (GDS) [13]. Neuropathological evaluation was performed on formalin fixed, paraffin embedded tissue from different sites, including the frontal cortex (F2), temporal pole cortex, parietal cortex (superior and inferior lobule), occipital pole cortex and the hippocampus (essentially CA1 and entorhinal area of the parahippocampal gyrus). The distribution and the density of neurofibrillary tangles was determined in Bodian-stained sections, while senile plaques were stained with the methenamine silver method [14]. AD pathology was staged according to Braak and Braak [15]. Cases with mixed Parkinson's or Lewy body disease were excluded from the study. Demographic information, clinical status, neuropathological staging and APOE genotype are shown in Table 1.

**Table 1:** Clinical status, demographic information, disease duration, neuropathological staging and APOE genotype of control and AD cases used in this study.

Case	Diagnosis	Sex	Age (years)	Duration (years)	GDS	Braak	Brain weight (grs)	PMD (hours: minutes)	APOE genotype
1	CTRL	F	52		0	0	1268	6:50	3-3

2	CTRL	M	53		0	0	1341	14:25	3-3

3	CTRL	F	64		0	0	1169	8:35	4-2

4	CTRL	M	70		0	0	1560	7:45	4-3

5	CTRL	F	72		0	1	1296	6:45	4-3

6	CTRL	M	73		0	0	1267	24:45	3-3

7	CTRL	F	76		0	1	1212	4:50	3-2

8	CTRL	F	77		0	2	1213	9:15	4-3

9	CTRL	M	78		0	1	1467	5:35	4-3

10	CTRL	F	78		0	1	1047	6:30	4-3

11	CTRL	M	78		0	1	1332	6:55	3-3

12	CTRL	M	79		0	1	1334	7:40	4-3

13	CTRL	M	82		0	2	1468	13:35	3-3

14	CTRL	F	82		0	1	1280	5:30	3-2

15	CTRL	F	83		0	2	1102	7:45	3-3

16	CTRL	F	85		0	3	1199	9:00	4-3

17	CTRL	F	88		0	2	1152	5:40	3-3

18	CTRL	F	92		0	1	1031	7:15	3-2

19	CTRL	M	98		0	1	1203	8:40	3-3

20	AD	F	65	16	7	6	1115	5:40	3-3

21	AD	M	67	?	6	5	1170	5:22	4-4

22	AD	F	70	10	7	6	1020	4:30	3-3

23	AD	F	76	12	7	6	1170	5:30	4-3

24	AD	M	78	8	6	5	1298	7:45	4-4

25	AD	F	80	?	7	6	946	5:20	4-4

26	AD	M	80	9	5	6	1240	4:20	4-3

27	AD	F	82	7	7	5	1013	4:10	4-3

28	AD	F	83	5	5	4	1288	5:20	4-3

29	AD	F	83	3	6	5	1095	3:45	4-3

30	AD	F	86	10	6	4	1158	4:45	4-3

31	AD	F	87	2	7	3	976	3:55	4-2

32	AD	F	89	2	5	3	1212	4:10	3-3

33	AD	F	91	4	4	3	1161	9:35	4-3

34	AD	F	91	7	5	4	1101	3:45	4-3

35	AD	F	93	10	6	4	943	6:15	3-3

36	AD	F	94	4	5	2	1050	3:15	3-3

37	AD	F	94	7	6	2	987	6:35	3-3

38	AD	F	97	7	7	3	1016	7:00	3-3

CTRL, control case; AD, Alzheimer's disease patient; F, female; M, male; GDS, Global Deterioration Scale; gr, gram; PMD, post-mortem delay.

### Immunocytochemistry

For immunohistochemical staining, formalin fixed (4%, 24 h) paraffin embedded tissue from mid-temporal cortex was used. Sections (5 μm thick) were mounted on superfrost-plus tissue slides (Menzel-Gläser, Germany) and deparaffinized. Subsequently, sections were immersed in 0.3% H_2_O_2 _in methanol for 30 min to quench endogenous peroxidase activity, and treated in 10 mM pH 6.0 citrate buffer heated by microwave during 10 min for antigen retrieval. Normal serum and antibodies were dissolved in phosphate-buffered saline (PBS) containing 1% (w/v) bovine serum albumin (BSA, Boehringer Mannheim, Germany). Sections were pre-incubated for 10 min with normal rabbit serum (DAKO, Glostrup, Denmark). Primary antibodies were incubated for 1 hr at room temperature. See Table 2 for antigen, dilution and source of primary antibodies. After washing with PBS, slides were incubated with biotin-conjugated rabbit anti-mouse antibody (rabbit anti-mouse F(ab')2, 1:500 dilution, DAKO) for 30 min. Subsequently, slides were incubated with streptavidin-biotin horseradish peroxidase complex (streptABComplex/HRP, 1:200 dilution, DAKO) for 60 min. Color was developed using 3,3'-diaminobenzidine (0.1 mg/ml, 0.02% H2O2, 3 min) as chromogen. Sections were mounted with Entellan (Merck, Darmstadt, Germany).

**Table 2:** Primary antibodies used in this study.

Antibody	Species	Antigen	Dilution	Source
KP1	mouse	CD68	1:400	Dako, Glostrup, Denmark

CR3/43	mouse	HLA-DP/DQ/DR	1:50	Dako, Glostrup, Denmark

GFAP (clone 6F2)	mouse	GFAP	1:10	Monosan, Uden, The Netherlands

AT8	mouse	Tau pSer202 and pThr205	1:1000	Pierce, Rockford, IL, USA

Aβ1-17	mouse	Aβ1-17	1:50	Dako, Glostrup, Denmark

### Quantitative analyses

For each case an area between the top and the depth of a gyrus was selected at random. Contiguous microscopic fields from the pial surface to the boundary with white matter perpendicularly to the axis of a gyrus made up a column. At least three columns were assessed per individual case. AT8-immunoreactive neurofibrillary tangles were counted using a 40 × objective (0.16 mm^2^). Aβ- and AT8-positive plaques were counted using a 10 × objective (0.64 mm^2^). In order to measure the amount of immunoreactivity for KP1, CR3/43 and GFAP, the area density of immunoreactivity was measured [4]. Contiguous microscopic fields arranged in columns were examined with a 10 × objective. Full color images were obtained using a Zeiss light microscope equipped with a digital camera. The area density was quantified using Image-Pro Plus analysis software (Media Cybernetics, Silver Spring, MD). Using this method the percentage of the area of interest that is immunoreactive for a specific antibody is measured. Assessments for different antibodies were performed in adjacent sections and blind to the pathological and clinical categorization.

### Statistical analyses

The Kruskall-Wallis test was used to evaluate differences between groups followed by the Mann-Whitney U test, to test differences between pairs of groups. Linear regression analysis was performed to model the relation between age and different variables in controls and AD cases. Beta coefficients of the relation between age and different variables were compared between control and AD cases. A p value < 0.05 was taken as significant.

## Results

**Figure 1:**
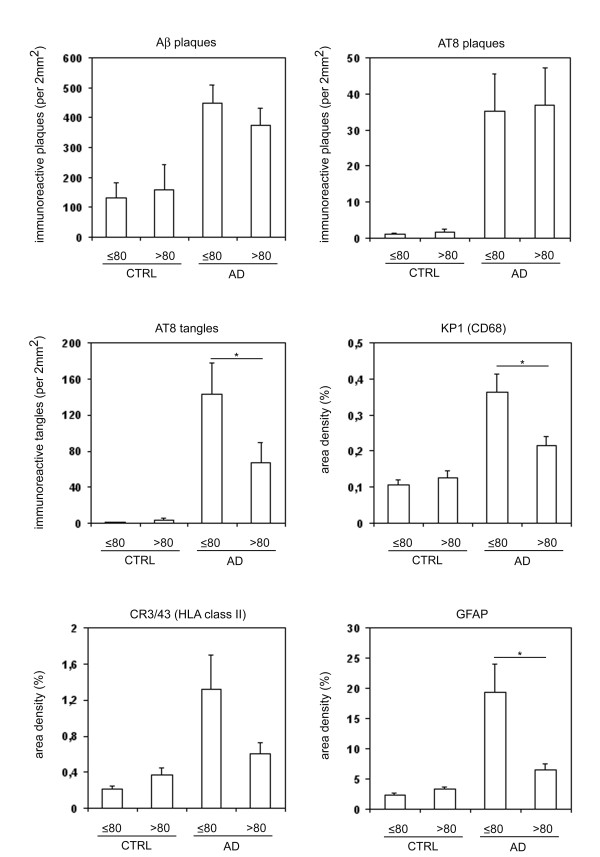
**Relative occurrence of pathological hallmarks and neuroinflammation in controls and AD cases**. Data represent the occurrence (Y axis in number per 2 mm^2^) of Aβ immunoreactive plaques, AT8 immunoreactive plaques and tangles, and the relative occurrence (Y axis in percentage) of quantified KP1 (CD68), CR3/43 (HLA class II), and GFAP immunoreactivity in control (CTRL) and AD cases of 80 years and younger (≤ 80) or older than 80 years (> 80). Data are shown as mean level ± S.E.M.. * indicates significant difference.

The relative occurrence of AD pathological hallmarks and neuroinflammation was assessed in two age-groups. A post-mortem age of 80 years was used as a cut-off as it provided comparable group sizes. The number of Aβ-immunoreactive plaques and AT8-positive plaques and tangles were determined in each case. For assessment of KP1, CR3/43 and GFAP immunoreactivity, the area density was determined and expressed as the percentage of area positive for each specific marker (Figure 1). For all markers there were significant differences between AD and control cases in the group of 80 years and younger, as well in the group older than 80 years. Except for CR3/43, no significant difference between control and AD cases could be observed in the group older than 80 years. Within the AD group, significant differences in AT8-positive tangles, KP1 and GFAP were observed between AD cases older and younger than 80 years. This data indicates that, in contrast to Aβ deposits and AT8 positive plaques, the occurrence of tangles, microglia and astrocytes is lower in old AD cases (> 80 years) compared to younger AD cases.

**Figure 2:**
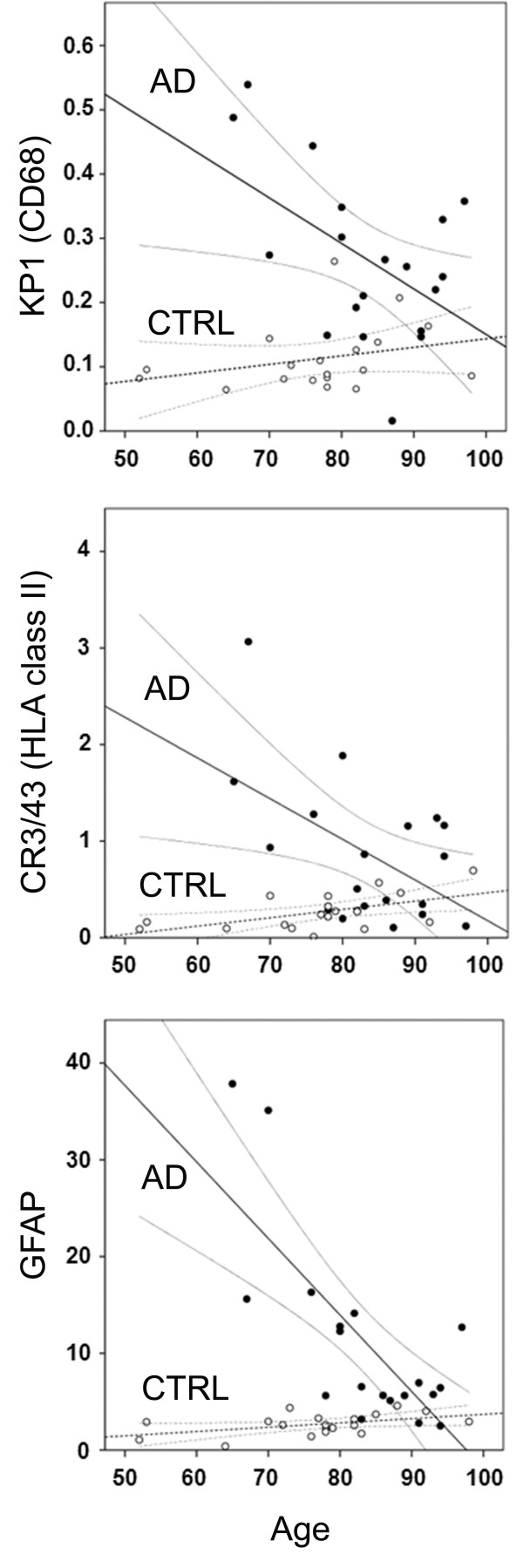
**Neuroinflammation in relation to age in controls and AD cases**. Data represent the relation between age (X axis, in years) and quantified KP1 (CD68), CR3/43 (HLA class II), and GFAP immunoreactivity (Y axis, in arbitrary units) for controls (open dots) and AD patients (closed dots). Straight lines represent regression lines, with corresponding 95% confidence intervals.

To study more directly the association between age of death and the area density of KP1, CR3/43 and GFAP in AD cases and controls, regression analysis was performed. The occurrence of KP1, CR3/43 and GFAP rapidly decreases with age in AD cases, in contrast to control cases (Figure 2). For all markers the 95% confidence intervals of control and AD cases start to overlap between post-mortem ages of 85 and 90 years. A significant beta-coefficient for regression over age was observed for KP1, CR3/43 and GFAP in AD cases. Comparison of respective beta-coefficients for regression over age confirmed significant differences between AD and control cases for KP1, CR3/43 and GFAP (Table 3). These data indicate that the occurrence of microglia and astrocytes decreases over age in AD, in contrast to control cases, suggesting that the association between neuroinflammation and AD is stronger in cases with relatively young age.

**Table 3:** Beta-coefficients of the regression of the age-dependent scores for Aβ deposits, AT8-positive neuritic plaques, AT8-positive neurofibrillary tangles, KP1, CR3/43 and GFAP immunoreactivity for control and AD cases.

	Beta-coefficient (Standard Error)		
**Variable**	**CTRL**	**AD**	**p-value**

Aβ deposits	5.411 (3.672)	-2.611 (4.845)	0.193

AT8 NPs	0.069 (0.033)*	0.153 (0.831)	0.912

AT8 NFTs	0.180 (0.062)*	-2.416 (2.247)	0.212

KP1	0.001 (0.001)	-0.007 (0.003)*	0.006

CR3/43	0.009 (0.003)	-0.042 (0.017)*	0.003

GFAP	0.044 (0.020)*	-0.791 (0.173)*	0.000

NPs, neuritic plaques; NFTs, neurofibrillary tangles; CTRL, control case; AD, Alzheimer's disease cases. P-value indicates difference between regressions of the AD and control groups. * indicates significant beta-coefficient.

Apolipoprotein E (apoE) has been shown to play a role in the innate immune response, and inheritance of the APOE4 allele is associated with increased risk of developing AD [16,17]. The APOE genotype is indicated in Table 1. In this study, the incidence of APOE4 in control cases younger than 80 years is 29% compared to 7% in older control cases. For AD cases, the incidence of APOE4 in cases younger than 80 years is 57% compared to 29% in older AD cases.

## Discussion

In this study, we compared the age-dependent presence of microglia and astrocytes, which is indicative of a neuroinflammatory response, in controls and AD cases. We show that the association between neuroinflammation and AD is stronger in relatively young AD cases compared to old AD cases. The difference in occurrence of these neuroinflammatory markers between AD and control cases decreases over age. Non-demented controls and AD cases were selected from sequentially performed autopsies over a period of two years. Inclusion criteria for controls included no reported signs of cognitive impairment during life. The presence of neurofibrillary tangles or neuritic plaques after neuropathological examination was not used as an exclusion criteria for controls (see Braak stage, Table 1). Inclusion criteria for AD cases were based on clinical signs of AD-type dementia. Dementia status at death was determined on the basis of all information available for each case during the last year of life. Severity of dementia was measured using the Global Deterioration Scale (GDS), which encompasses activities of daily living, behaviour and cognition, and is not affected by the educational level of patients [13]. In using the above mentioned inclusion criteria and sequential selection over a period of two years we sought to avoid possible bias related to age and severity of the underlying pathology that causes the disease.

Previous reports have indicated that activation or presence of microglia as well as the occurrence of gliosis increases with normal aging [18-20]. In the present study a significant beta-coefficient was observed in control cases for GFAP (Table 3), indicating an increased occurrence of astrocytes with normal aging. Although levels of microglia appear to increase slightly with aging in controls, no significant changes were observed. Probably, the statistical power of the current study is too low to observe a significant increase in microglia over age in controls. Other explanations, like differences in detection technique and markers, cannot be excluded. The previously observed increased occurrence of these markers for microglia and astrocytes with normal aging suggests that these markers could be considered as markers for senescence rather than for inflammation. This suggests the rather controversial idea that the high expression levels of these markers in younger AD patients are a marker of 'exacerbated glial senescence' at relatively young ages that could be involved in the pathogenesis of AD. Another interpretation of this data could be that successful aging, i.e. without dementia, is associated with an increased presence and function of microglial cells, due to the increasing demand on the neuro-supportive and neuroprotective roles of microglia [21]. There are indications that microglia have a role in neurite development and it is hypothesized that microglia regulate synapse physiology [22]. The increase in microglia in young AD cases might reflect a regenerative response [23], which might become less effective with increasing age in AD. The connection between microglia and neuronal function is reflected in the significantly higher number of neurofibrillary tangles in AD patients younger than 80 years compared to older AD patients (Figure 1).

The observed decrease in neuroinflammation in the AD group could reflect a selective loss of glial cells with the aging process. It has been observed that microglial cells in aged human brain are dystrophic, showing morphological features indicative of senescence and degeneration, such as cytoplasmic processes [24]. Association of senescent microglia with tau pathology in AD may be interpreted as an indirect sign of the age-dependent failing of the neuro-supportive and neuroprotective roles of microglia that contribute to the neurodegenerative process in AD [12,21,24]. Determination of microglia senescence requires a more detailed qualitative morphological assessment. The quantification method used in this study did not assess microglial morphology, which is required for this phenotypic characterization. In addition, the markers used in the current study are those generally used for the detection of microglia and are not indicative of microglial activation state [25-28], thereby not reflecting the dual role of microglia in the inflammatory response in AD [29,30]. Future studies are needed to address activation state and phenotypic morphology, as well as selective loss of glial cells in AD in relation to aging.

Inheritance of APOE4, the gene that encodes apolipoprotein E4, is a major risk factor for late onset AD. The incidence of the APOE4 allele in the general population is 20-25%, whereas the incidence in patients with AD rises to 50-65%. The presence of at least one APOE4 allele has been associated with earlier age at onset [17]. The hypothesised frequency distribution of the association between age at onset and the presence of APOE4 would therefore suggest that the occurrence of APOE4 decreases with age in people with AD [31]. The apoE protein is an immunomodulatory protein that affects both innate and adaptive immune responses. Microglia derived form targeted replacement mice carrying two copies of the APOE4 allele show a pro-inflammatory phenotype compared to microglia derived from mice carrying two copies of the APOE3 allele [16]. In the present study the incidence of APOE4 in AD cases of 80 years and younger was 57%, while the incidence of APOE4 in AD cases older than 80 years was much lower, at 29% (Table 1). This decreased frequency of APOE4 alleles could explain the difference in occurrence of neuroinflammation between young and old AD patients, and supports the hypothesis that a proinflammatory genetic profile contributes to an earlier onset of AD.

Inflammation has been advanced as one of the underlying mechanisms that drives AD pathology; however, clinical trials with anti-inflammatory drugs have generated inconclusive results [32]. The timing of anti-inflammatory treatment is crucial since clinical studies have indicated that inflammation occurs in early stages of AD, perhaps even before exceeding the clinical detection threshold [9,10]. Data from the current study would imply that age itself could be an important factor for the response to therapy since the occurrence of inflammation in AD dementia decreases with increasing age of death. In addition, we show that the occurrence of microglia and astrocytes in AD and non-demented cases starts to overlap between post-mortem ages of 80 and 90 years. This data is in line with earlier studies which report considerable overlap in the severity of neurofibrillary tangle and neuritic plaque pathology between older patients with AD dementia and non-demented control cases [33,34]. The study of Savva et al. implies that the diagnostic value of biomarkers related to the underlying biological process leading to accumulation of tangles or plaques is much more prominent in the relatively young compared with the oldest patients, and indicates that the pathological substrate that causes dementia is heterogeneous and age-dependent [33]. The age-dependent relationship between the underlying biology and clinical phenotype of AD has implications for the interpretation of biomarkers and treatment of the disease. Data from the current study implicates the importance of considering age when interpreting the results of studies on inflammatory biomarkers for AD.

## Competing interests

The authors declare that they have no competing interests.

## Authors' contributions

JJMH, PE and WAvG designed the study. JJMH coordinated the study and was responsible for writing the manuscript. ESvH carried out most of the lab work and analyzed the data. PE and WAvG participated in writing the manuscript. AJMR was responsible for the autopsy material and neuropathological evaluation. All authors read and approved the final manuscript.
